# Delayed Right Hemidiaphragm Rupture After Blunt Trauma Repaired With Prolene Mesh: A Case Report

**DOI:** 10.1155/cris/3439339

**Published:** 2026-09-23

**Authors:** Zahra Sadin, Aghajanzadeh Manochehr, Sadin Mohammadreza, Alavi Foumani Ali, Fakhr Mousavi Ali Asghar, Mosaffaee Omid, Ramezani Naghi, Farzin Mohaya

**Affiliations:** ^1^ Department of Thoracic Surgery, Guilan University of Medical Sciences, Rasht, Iran, gums.ac.ir; ^2^ Department of Pulmonology, Guilan University of Medical Sciences, Rasht, Iran, gums.ac.ir; ^3^ Department of Internal Medicine, Inflammatory Lung Diseases Research Center, Razi Hospital, School of Medicine, Guilan University of Medical Sciences, Rasht, Iran, gums.ac.ir

**Keywords:** blunt trauma, delayed presentation, diaphragmatic rupture, Prolene mesh, right hemidiaphragm

## Abstract

Traumatic diaphragmatic rupture (TDR) is a rare but serious injury. It most commonly results from high‐impact blunt trauma. Right‐sided ruptures are particularly uncommon due to the protective effect of the liver. Delayed presentation can cause diagnostic challenges, as the injury may remain clinically silent for years before complications such as organ herniation. We report a case of a 65‐year‐old male who presented with progressive dyspnea, right‐sided chest pain, and right upper quadrant abdominal discomfort 4 years after a severe motor vehicle accident. Chest radiography revealed a heterogeneous opacity in the right hemithorax, and contrast‐enhanced CT confirmed right hemidiaphragmatic rupture with intrathoracic herniation of the liver. The patient underwent urgent surgical repair. A large diaphragmatic defect was identified and repaired with a Prolene mesh in a tension‐free fashion. The postoperative course was uneventful, with complete resolution of symptoms. This case highlights the importance of maintaining clinical suspicion for delayed diaphragmatic rupture in patients with a remote history of blunt trauma, even when initial imaging was normal. Contrast‐enhanced CT remains the diagnostic standard, and Prolene mesh repair is a surgical solution for large diaphragmatic defects.

## 1. Introduction

Traumatic diaphragmatic rupture (TDR) is a rare but serious injury, most often caused by high‐impact traumas such as motor vehicle accidents. It is reported in 0.8%–5.8% of blunt thoracoabdominal trauma cases. Right‐sided ruptures are less common than left‐sided injuries because the liver distributes the traumatic force and because right‐sided defects are more easily overlooked on initial imaging before herniation develops [1–3]. A distinctive feature of these injuries is their potential to remain clinically silent for years and only become apparent when complications such as organ herniation develop. Like when the liver or stomach protrude into the chest and cause symptoms like chest pain or shortness of breath [1, 4]. This latency can reflect a small initial tear that is temporarily sealed by the adjacent liver or omentum, and frank herniation only occurs later when intra‐abdominal pressure rises from heavy lifting or another Valsalva‐type maneuver. It forces the abdominal contents through the defect. Diagnosing a delayed rupture is not easy: chest X‐rays can miss up to 25% of cases, so contrast‐enhanced CT scans remain the preferred method for detecting defects and herniated organs [5, 6]. In our patient, a 4‐year delay from a car accident to the onset of symptoms shows how these injuries can remain silent and can only present when physical exertion triggers complications. Regarding repair of large TDR, primary closure is not always possible, especially for defects larger than 8–10 cm; in such cases, synthetic meshes such as Prolene provide a tension‐free repair [3, 5]. Biologic meshes are also an option, but there is not enough evidences of their superiority over synthetics [2, 5, 7]. This case report explores a rare delayed right hemidiaphragm rupture with liver herniation, which was managed with Prolene mesh repair. It highlights the diagnostic challenges and surgical considerations in managing large TDRs [1, 3].

## 2. Case

A 65‐year‐old male presented to the pulmonary medicine department of a teaching hospital with complaints of severe right‐sided chest pain, right upper quadrant abdominal discomfort, progressive dyspnea, constipation, decreased appetite, and a productive cough lasting for 12 days from the onset. These symptoms began several days after the patient lifted a heavy package.

On examination, the patient was afebrile but in respiratory distress, with a respiratory rate of 34 breaths per minute, an oxygen saturation of 96% on room air, a pulse rate of 96 beats per minute, and a blood pressure of 130/80 mmHg. Abdominal examination revealed a flat and tender abdomen, most notably in the right upper quadrant. On chest examination, breath sounds were decreased in the mid and lower zones of the right lung, and cardiac auscultation revealed a shift of the point of maximal impulse to the left hemithorax. It was consistent with mediastinal displacement secondary to right‐sided diaphragmatic herniation.

**Figure 1:**
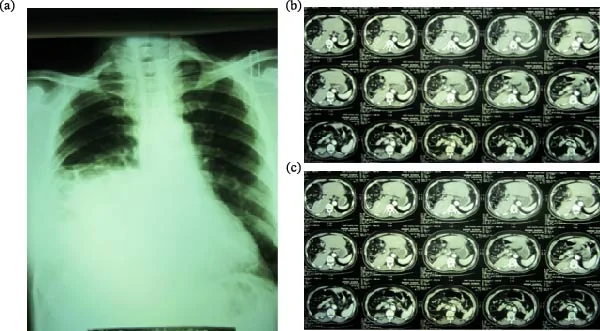
Preoperative imaging findings. Posteroanterior chest radiograph demonstrating a heterogeneous opacity in the right hemithorax, raising initial suspicion for pleural or pulmonary pathology (a). Contrast‐enhanced CT scan of the thorax (axial view) confirming right hemidiaphragmatic rupture with intrathoracic herniation of the liver (b). Coronal CT reconstruction illustrating the extent of liver displacement into the right thoracic cavity (c).

A posteroanterior chest radiograph showed a heterogeneous opacity in the right hemithorax (Figure 1). It initially raised suspicion of a pleural or pulmonary pathology. Abdominal ultrasonography revealed the liver vertically within the right hemithorax. It suggests herniation through the diaphragm. A contrast‐enhanced CT scan of the thorax and abdomen confirmed the diagnosis of right‐sided diaphragmatic rupture with intrathoracic herniation of the liver.

The patient had no known chronic cardiopulmonary disease or other significant comorbidities and was not on anticoagulant or antiplatelet therapy. He just reported a history of a severe motor vehicle accident about 4 years ago, and he was evaluated and discharged after an unremarkable initial trauma assessment. Medical records from that admission were not available for review. No follow‐up was done after that injury. Based on the clinical history, it was retrospectively suspected that a minor diaphragmatic tear had occurred during the initial trauma and remained undiagnosed. It was gradually enlarging over time and eventually led to complete rupture because of increased intra‐abdominal pressure by lifting the heavy object.

**Figure 2:**
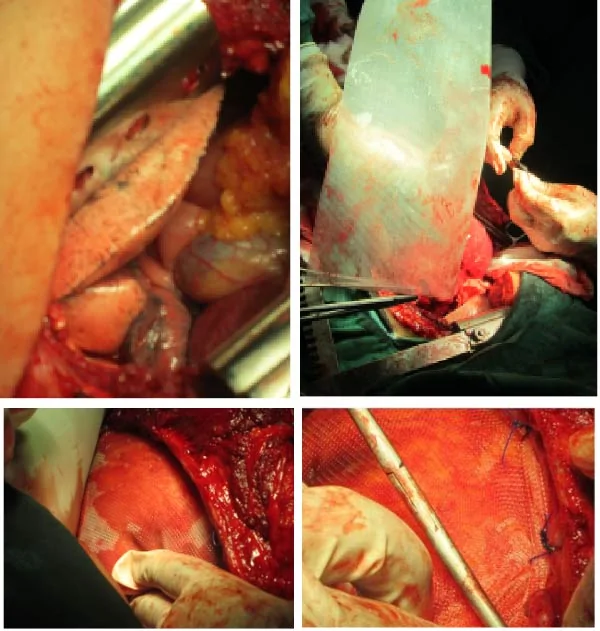
Intraoperative findings. Direct visualization of the large right diaphragmatic defect following thoracotomy. Herniation of the liver into the right thoracic cavity as seen intraoperatively. Intraoperative photograph showing the extent of hepatic displacement within the thorax prior to reduction. Complete reduction of the herniated liver with visualization of the diaphragmatic defect before mesh repair.

Following multidisciplinary consultation with thoracic surgery and pulmonary medicine, the patient was admitted for urgent surgical intervention by a right posterolateral thoracotomy. In operation, a large right diaphragmatic defect of about 10 cm × 8 cm was identified, with herniation of the liver into the thoracic cavity; the size of the defect prevents the tension‐free primary closure. So, it was the indication for prosthetic mesh reconstruction (Figure 2).

**Figure 3:**
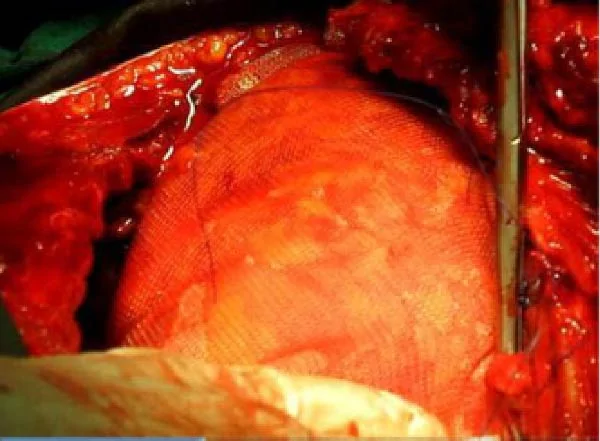
Repair of the large diaphragmatic defect after complete reduction of the herniated liver into the abdominal cavity, by a Prolene mesh.

**Figure 4:**
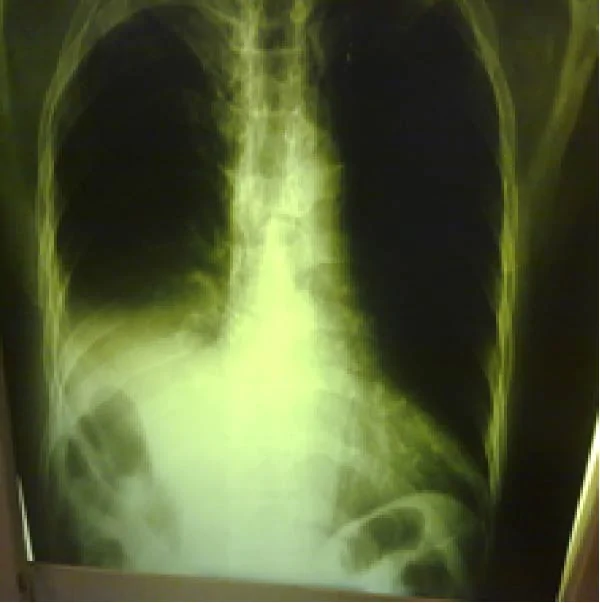
Chest X‐ray after surgery. Follow‐up imaging shows restoration of the normal anatomical position of the liver with resolution of the previous intrathoracic herniation and improvement of the right hemithoracic findings.

Complete reduction of the herniated liver was performed, and the residual diaphragmatic defect was repaired with a polypropylene (Prolene) mesh. It overlapped the defect margins by at least 2 cm and was secured circumferentially to the residual diaphragmatic rim and adjacent costal margin with interrupted and nonabsorbable 0‐Prolene mattress sutures. So, we made a tension‐free repair (Figure 3). The after surgery course was uneventful, with resolution of chest pain and dyspnea within 48 h. The patient was discharged on postoperative day 5 in stable condition. At the 2‐week follow‐up visit, he remained asymptomatic, with a normal chest radiograph and no clinical signs of mesh‐related complications; at the 3‐month visit, follow‐up chest imaging confirmed an intact repair with no recurrence of herniation and complete resolution of the previous right hemithoracic opacity (Figure 4).

## 3. Discussion

A diaphragmatic hernia is when abdominal organs protrude into the thoracic cavity through a diaphragmatic defect or rupture [8]. About 75% of such hernias are because of penetrating or blunt trauma, which results in diaphragmatic rupture [7, 8]. The incidence of diaphragmatic rupture ranges from 0.8% to 5% in cases of thoracoabdominal trauma [1, 3]. The simultaneous herniation of the liver, gallbladder, stomach, and omentum is still rare [5, 7].

Delayed diaphragmatic rupture may manifest days or weeks after the initial trauma, which permits abdominal organs to herniate into the thoracic cavity [5]. A diagnostic delay is common between the injury and the identification of complications [3]. Predominantly, 80%–90% of TDRs occur on the left side because of the vulnerability relative to the right diaphragm, which is reinforced by the liver. These injuries may initially go undetected due to concurrent trauma or nonspecific symptoms, and they only become evident later with complications such as organ incarceration and strangulation [3].

The majority of patients with diaphragmatic hernias are asymptomatic. When symptoms arise, they are influenced by the defect’s size, the organs involved, and any coexisting pulmonary conditions. Symptoms are abdominal pain, nausea, vomiting, dyspnea, chest pain, tachypnea, or cough. Strangulation particularly involving the colon or small intestine, so it can cause abdominal distention and acute abdominal symptoms. It carries the risks of morbidity and mortality [4].

The diagnosis has challenges. The initial findings of chest X‐rays are misinterpreted in 25% of cases [5]. The sensitivity of chest X‐rays varies, ranging from 2% to 60% for left‐sided hernias and 17%–33% for right‐sided hernias [3]. So, contrast‐enhanced CT scans are the diagnostic standard for confirming diaphragmatic hernias [1]. A recent multicenter analysis of 35 patients that were treated for TDR across three centers over a decade confirmed contrast‐enhanced CT as the diagnostic mainstay. It also mentions the persistent diagnostic delay with right‐sided TDRs [6]. Several previously reported cases of delayed right‐sided TDR have also been repaired with a mesh [1, 3, 4]. Three features, however, distinguish this case from those reports. First, the interval between trauma and presentation was 4 years, which was longer than the days‐to‐months latency described in most published delayed cases. This rupture was triggered by a specific mechanical event (heavy lifting) rather than a gradual onset. Second, herniation involved the liver alone, without the bowel, gallbladder, or omentum, which was in those reports. This case has isolated hepatic herniation without visceral strangulation, which is uncommon and prevents ischemic and obstructive complications that cause urgent presentation. Third, the defect was repaired in a single open procedure by a large Prolene mesh alone, without the dual‐mesh or staged reinforcement techniques that some prior reports required [3]. This patient had complete symptom resolution within 48 h and an intact repair at 3 months. Taken together, these features suggest that even very long‐standing and large right TDRs with isolated organ herniation can be managed successfully with a single‐stage mesh repair. This is the main point of the case.

Surgical intervention is still the primary approach to treatment. It repositions the herniated organs into the abdominal cavity and repairs the diaphragmatic defect. For smaller defects, primary closure with nonabsorbable sutures is adequate [4]. Larger defects that cannot be closed directly necessitate mesh reconstruction [3, 5]. In some cases, the diaphragm may require reattachment to the ribs at a higher‐than‐normal anatomical position. Reconstruction materials vary according to surgical preference. Synthetic meshes such as Prolene are widely utilized and generally well‐tolerated [3]. In the current case, the extensive defect required a complete Prolene mesh repair.

## Author Contributions

Zahra Sadin contributed to patient care, conceptualization of the study, drafting of the manuscript, and performed critical revision of the manuscript for important intellectual content. Aghajanzadeh Manochehr and Sadin Mohammadreza participated in patient management and data acquisition. Alavi Foumani Ali, Fakhr Mousavi Ali Asghar, Mosaffaee Omid, Ramezani Naghi, and Farzin Mohaya assisted in literature review and manuscript preparation.

## Funding

No funding was received for this study.

## Disclosure

All authors have read and approved the final version of the manuscript.

## Consent

Written informed consent was obtained from the patient for the publication of this case report and accompanying images.

## Conflicts of Interest

The authors declare no conflicts of interest.

## Data Availability

The data supporting the findings of this study are included within the article.
